# Cherenkov excited short-wavelength infrared fluorescence imaging *in vivo* with external beam radiation

**DOI:** 10.1117/1.JBO.24.5.051405

**Published:** 2018-11-22

**Authors:** Xu Cao, Shudong Jiang, Mengyu Jeremy Jia, Jason R. Gunn, Tianshun Miao, Scott C. Davis, Petr Bruza, Brian W. Pogue

**Affiliations:** a Dartmouth College, Thayer School of Engineering, Hanover, New Hampshire United States; b Xidian University, Engineering Research Center of Molecular and Neuro Imaging of the Ministry of Education, School of Life Science and Technology, Xi’an, Shaanxi, China

**Keywords:** Cherenkov emission, short-wavelength infrared, fluorescence imaging, radiation

## Abstract

Cherenkov emission induced by external beam radiation therapy from a clinical linear accelerator (LINAC) can be used to excite phosphors deep in biological tissues. As with all luminescence imaging, there is a desire to minimize the spectral overlap between the excitation light and emission wavelengths, here between the Cherenkov and the phosphor. Cherenkov excited short-wavelength infrared (SWIR, 1000 to 1700 nm) fluorescence imaging has been demonstrated for the first time, using long Stokes-shift fluorophore PdSe quantum dots (QD) with nanosecond lifetime and an optimized SWIR detection. The $1/\lambda^{2}$ intensity spectrum characteristic of Cherenkov emission leads to low overlap of this into the fluorescence spectrum of PdSe QDs in the SWIR range. Additionally, using a SWIR camera itself inherently ignores the stronger Cherenkov emission wavelengths dominant across the visible spectrum. The SWIR luminescence was shown to extend the depth sensitivity of Cherenkov imaging, which could be used for applications in radiotherapy sensing and imaging in human tissue with targeted molecular probes.

Cherenkov emission induced by external beam radiation therapy (EBRT) from a clinical linear accelerator (LINAC) produces internal Cherenkov light within tissue, and can excite phosphors or fluorophores. The emitted luminescence can be used to image molecular signatures of tumor in deep tissue during radiation therapy.^1^^,^^2^ In this study, the use of short-wavelength infrared (SWIR) emitting agents was examined.

The external radiation beam delivered from a LINAC is a 3.5-μs temporal square pulse with a repetition rate of 360 Hz. Using this irradiation method, Cherenkov excited luminescence imaging (CELI) can be achieved using a phosphor and time-gated detection to isolate the luminescence signal from the Cherenkov excitation signal. Previous work showed that quantitative partial pressure of oxygen (${\mathrm{pO}}_{2}$) imaging of tumor microenvironment could be achieved with the phosphor sensor Platinum (II)-G4 (PtG4).^3^ However, most optical probes are fluorophores with nanosecond lifetimes, and so are much shorter than the radiation pulse. Thus, time-gated detection cannot be effectively used to acquire fluorescent signals when excited by Cherenkov emission, which severely limits the applications of CELI in biological and medical imaging. The spectral emission and fitting of fluorophores could be detected,^4^^,^^5^ but ideally, the fluorophore would have an emission spectrum as far out in wavelength as possible to maximize the signal to background.

To achieve this goal, Cherenkov excited SWIR (1000 to 1700 nm) fluorescence imaging (CESFI) was demonstrated using fluorophore PdSe quantum dots (QDs) (Fig. 1). Due to the $1/\lambda^{2}$ characteristic of the Cherenkov emission spectrum,^6^ the proposed CESFI approach can effectively collect SWIR fluorescent signals by minimizing the overlapped Cherenkov emission based on the characteristic of a long wavelength interval between excitation and emission. Other attractive advantages of SWIR light such as high resolution and deep penetration are benefits from weaker scattering as it propagates through biological tissues.^7^^–^^9^ CESFI could also provide scanned sensing, for high sensitivity imaging with high spatial resolution for molecular sensing in preclinical applications.

**Fig. 1:**
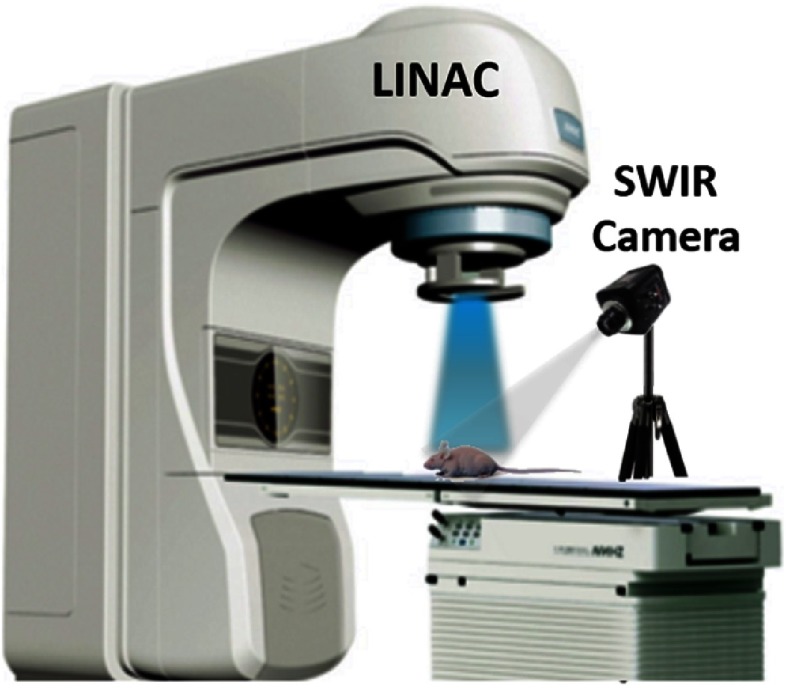
Imaging setup for CESFI with an electron beam (blue).

As shown in Fig. 1, a Varian LINAC (Varian LINAC 2100C, Varian Medical Systems, Palo Alto) was employed to generate the megavoltage electron beam and irradiate the target in a vertical direction. A SWIR InGaAs camera (NIRvana 640 Princeton Instruments, Acton) was coupled with a Canon 135 mm f/2.0 lens and fixed on a tripod about 2 m away from this object. All ambient light sources in the LINAC room were turned off when data were acquired.

Figure 2 shows the absorption and emission spectra of the PdSe QD and spectrum of Cherenkov emission normalized with their corresponding maximum values. The black dashed curve is the theoretical spectrum of Cherenkov emission from 200 to 1500 nm, as calculated by the Frank–Tamm formula. As shown in the blue dash-dot curve, the absorption of PdSe QD rapidly decreased from shorter to longer wavelengths, below 300 nm. The absorption was cut off at 200 nm due to the lower limit of the fluorescence spectrophotometer. The UV/blue spectral distributions of Cherenkov emission match well with the absorption of PdSe QD, so that Cherenkov emission should be able to excite PdSe QD effectively. The emission spectrum of PdSe QD (red solid curve) has a peak at 1030 nm.

**Fig. 2:**
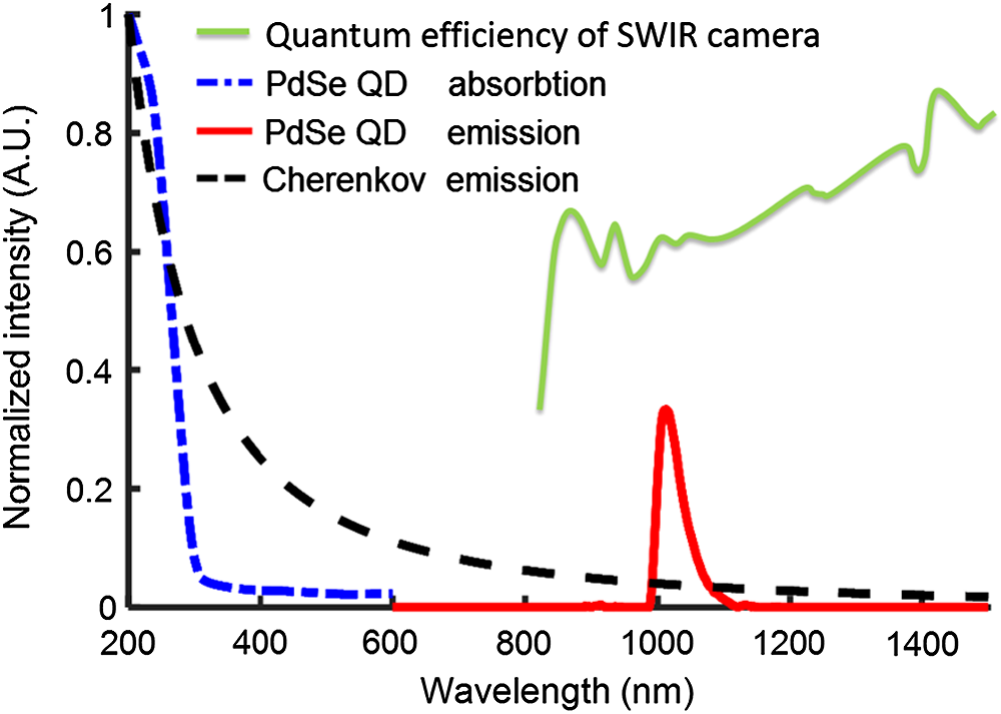
Absorption and emission spectrum of PdSe QD and SWIR camera spectral sensitivity.

For the phantom experiment, a tube filled with 200  μL PdSe QD at concentration of 100  μM was used as the target object. As shown in Fig. 3(a), the tube was fixed on the edge of a water tank at a depth of 1 cm under the water. A 6 MeV electron beam with filed size of $5\times 1\;{\mathrm{cm}}^{2}$ was projected into the water tank from the top over the tube, and the SWIR camera was used to acquire Cherenkov excited fluorescence. Fluorescein, a commonly used fluorophore with high quantum yield, was used with the same concentration and volume as a comparison target object. A Pi-Max 3 ICCD camera, which is sensitive to visible and near infrared wavelength region (Vis-NIR, 400 to 900 nm), was employed to acquire Cherenkov excited fluorescence of fluorescein. To compare with time-gated acquisition, PtG4 with volume of 200  μL at concentration of 100  μM was also imaged. The Pi-Max 3 ICCD camera can be triggered by the falling edge of square pulse with a 5  μs delay, which was employed to ensure the acquisition of phosphorescence without Cherenkov emission from the electron beam.

**Fig. 3:**
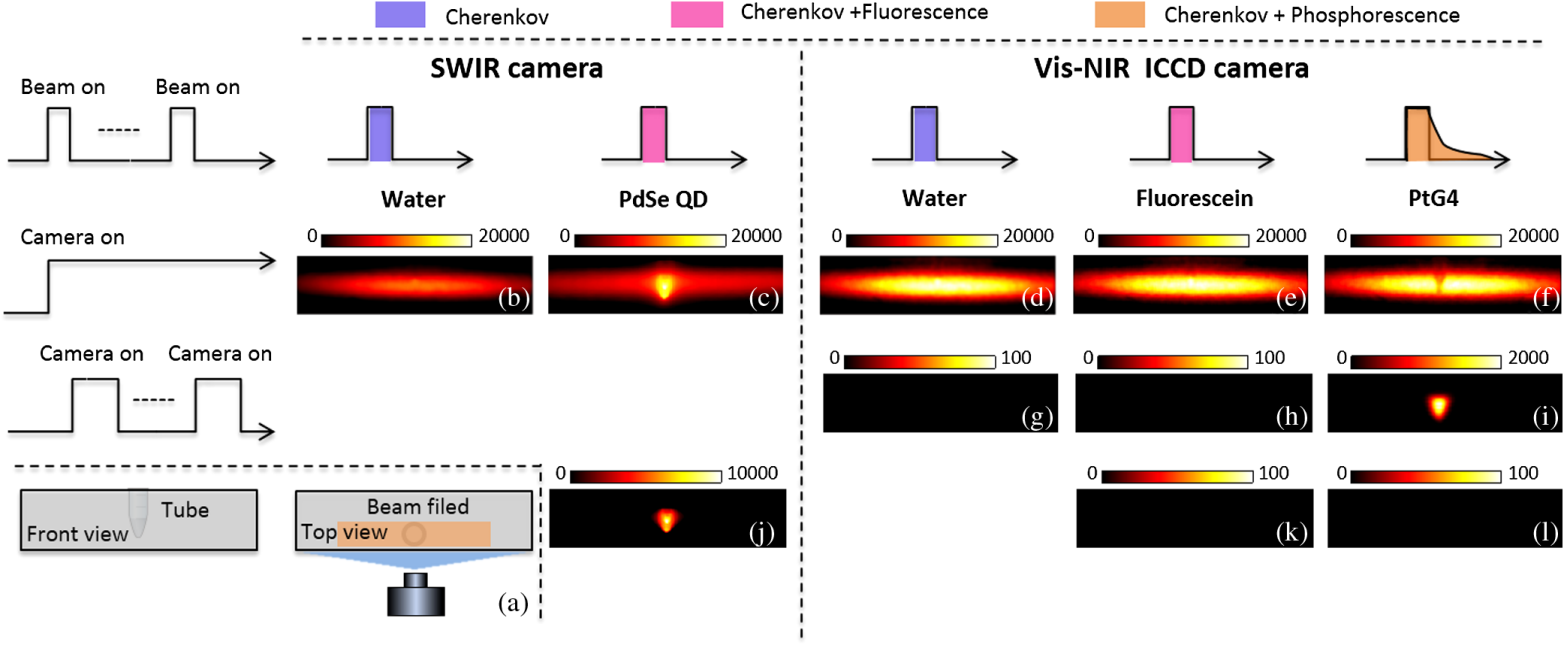
Phantom study setup and images. (a) Experimental setup. (b–f) Images obtained without time-gated acquisition. (g–i) Images obtained with time-gated acquisition. (j) Image of PdSe QD by subtracting Cherenkov emission background (b) from (c). (k) Image of fluorescein by subtracting Cherenkov emission background (d) from (e). (l) Image of PtG4 by subtracting Cherenkov emission background (d) from (f).

Figures 3(b)–3(f) show the images of water [Figs. 3(b) and 3(d)], PdSe QD [Fig. 3(c)], fluorescein [Fig. 3(e)], and PtG4 [Fig. 3(f)] without time-gating. The SWIR or Pi-Max 3 ICCD camera was triggered when the electron beam was on, using exposure times of 10 and 0.3 s, respectively. Due to the scattering of electrons, the Cherenkov emission was highly diffused in water [Figs. 3(b) and 3(d)]. As shown in Fig. 3(c), the PdSe QD tube was clearly observable in the center of the image with the Cherenkov emission background over the entire radiation beam area. In contrast, the fluorescence/phosphorescence of fluorescein and PtG4 was completely obscured by the Cherenkov emission background [Figs. 3(e) and 3(f)]. The shading at the location of the tube was due to the absorption of PtG4 from the Cherenkov emission.

Using time-gated acquisition, the Pi-Max 3 ICCD camera could negate all of Cherenkov emission background, as shown in Fig. 3(g). Since the lifetime of fluorescein is only several nanoseconds, the time-gated acquisition approach could not be used to collect any signal from fluorescein [Fig. 3(h)]. Due to the long lifetime of PtG4, the Cherenkov emission background was completely removed by time-gated acquisition in Fig. 3(i).

Figures 3(j), 3(k), and 3(l) are images of PdSe QD, fluorescein, and PtG4 after subtracting the Cherenkov emission background from Figs. 3(c), 3(e), and 3(f), respectively. PdSe QD was displayed clearly in Fig. 3(j), but the entire radiation areas were zero fluorescence/phosphorescence for fluorescein [Fig. 3(k)], and PtG4 [Fig. 3(l)]. Comparing Figs. 3(j) and 3(i), it can be seen clearly that fluorescence imaging without time-gated acquisition can be obtained in SWIR range with similarly high quality to that seen from phosphorescent imaging with time-gated acquisition.

To test the feasibility of *in vivo* CESFI, 100-μL PdSe QD with concentration of 100  μM mixed with 100  μL matrigel was injected subcutaneously into a nude mouse on its back. Then, the nude mouse was anesthetized using isoflurane and placed prone on a black surface for imaging. A warming pad was used to keep the temperature of the mouse during imaging. Similar to the phantom imaging, a 6-MeV electron beam shaped by the collimator into a 10×20  mm filed size was delivered to the mouse. The SWIR camera exposed for 10 s to acquire images when the electron beam was on. White light image was also obtained with room light on but without the electron beam.

Figure 4(a) shows the white light image of the mouse obtained by the SWIR camera. Figure 4(b) is a mixture of SWIR Cherenkov emission and the SWIR fluorescence of PdSe QD, as obtained by the SWIR camera when the mouse was irradiated with a 6-MeV electron beam. The SWIR Cherenkov emission was distributed over the whole body of the mouse. However, the counts for PdSe QD containing location are higher than the areas without them, and so, the contrast was observed at 1.4. A rectangular region 1 in Fig. 4(b) without PdSe QD was selected for estimation of Cherenkov emission background. After subtracting the average of this region from the image in Fig. 4(b), the SWIR fluorescence of PdSe QD was obtained, as shown in Fig. 4(c). Figure 4(d) shows the ratio between Cherenkov emission background (average of region 1) and estimated SWIR fluorescence of PdSe QD (average of region 2).

**Fig. 4:**
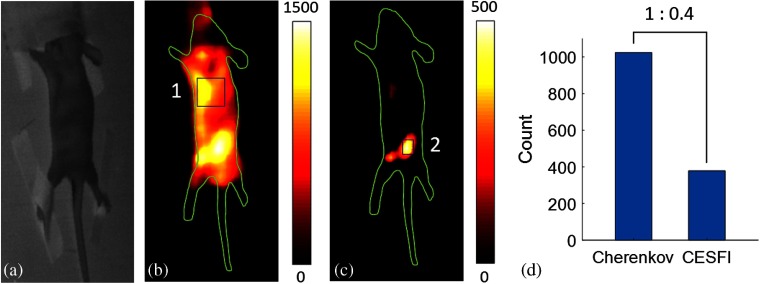
*In vivo* study. (a) White light image of a mouse. (b) CESFI image of a mouse with subcutaneous injection of PdSe QD. (c) Image after background Cherenkov subtraction. (d) Intensity ratio between Cherenkov emission marked in square region 1 and SWIR fluorescence of PdSe QD marked in square region 2.

Generally, a fluorescent probe absorbs a short wavelength excitation light and emits a long wavelength fluorescent light. If the intensity of emitted fluorescent light is weaker than that of Cherenkov light at the emission wavelength of the fluorescent probe, it is impossible to separate useful fluorescent signal from the Cherenkov emission. Since the intensity of Cherenkov emission is decreasing with the square of wavelength, so a large Stokes shift (between excitation wavelength and emission wavelength) results in much weaker Cherenkov signal at the emission wavelength of the fluorophores, comparing to that at the excitation wavelength. The chosen PdSe QD agent has a characteristic of UV wavelength excitation and SWIR wavelength emission, which leads to effective excitation with low overlap. Although Cherenkov emission has components in the SWIR range, the fluorescence of PdSe is comparable in magnitude to the SWIR Cherenkov emission, and so it can be effectively recovered with appropriate background subtraction methods.

For fluorescent imaging, a notch filter around the excitation wavelength is usually used to filter out the excitation light. In the present study, since the SWIR camera itself acts as a long pass filter, eliminating the excitation light below 900 nm (because the SWIR camera has no sensitivity below 900 nm), no filter was used to eliminate the exciting Cherenkov light for any of the data acquisition. Considering that Cherenkov emission still exists in the SWIR range and the emission of a SWIR fluorescent probe is usually concentrated in a narrow wavelength bandwidth, a band-pass filter around the fluorescent emission wavelength could further improve the signal-to-noise ratio of CEFSI. Since the integral intensity of SWIR Cherenkov emission is about 10 times higher than that of the overlapping Cherenkov emission (Fig. 2), by using a bandpass filter with center wavelength of 1030 nm and bandwidth of 40 nm, it would be possible to achieve 10 times higher signal-to-noise ratio for CEFSI.

The camera noise due to the radiation was proportional to the square of the distance between the SWIR camera and the LINAC. After this series of tests, a long imaging distance of 2 m was found to be the optimal distance to obtain maximum signal-to-noise ratio for the present camera shielding conditions. Through the use of using better shielding, superior spatial resolution could be expected by moving the camera much closer to LINAC and with a higher f number lens.

This recently developed approach for Cherenkov excited luminescence scanned imaging used a thin megavoltage x-ray beam to scan an object with a light sheet approach, to acquire three-dimension (3-D) optical imaging of molecular tracers with submillimeter resolution and nanomolar sensitivity.^10^ Due to lower scattering in the SWIR wavelengths, as compared to the Vis-NIR wavelength range, higher spatial resolution images at deeper tissue penetration depths could be expected for biological imaging *in vivo*. High quality 3-D *in vivo* optical molecular imaging based on CESFI with the light sheet scanning could be implemented in future work.
